# Genomic epidemiology of *Brucella suis* biovar 2 in German swine and wildlife, 2003–2023

**DOI:** 10.3389/fvets.2025.1611681

**Published:** 2025-06-20

**Authors:** Falk Melzer, Jörg Linde, Hanka Brangsch

**Affiliations:** Institute of Bacterial Infections and Zoonoses, Friedrich-Loeffler-Institut, Jena, Germany

**Keywords:** brucellosis, *Brucella suis* biovar 2, domestic pig, wild boar, hare, cgMLST, cgSNP

## Abstract

**Introduction:**

Porcine brucellosis, caused by *Brucella suis* biovar 2, is currently the only type of brucellosis officially reported in farm animals in Germany, with outbreaks confirmed through direct pathogen detection. In most European countries, this bacterial pathogen is also found in wild animals, which are considered reservoirs for the disease. Since 2003, 22 outbreaks of porcine brucellosis have been reported in Germany.

**Methods:**

A comprehensive study was conducted on German *B. suis* biovar 2 isolates obtained from routine diagnostic investigations of domestic pigs and wildlife. The dataset included isolates from 18 reported outbreaks. The aim was to assess epidemiological links and the genomic diversity of the bacterium. *B. suis* biovar 2 isolates were subjected to whole-genome sequencing (WGS) and analyzed using multilocus sequence typing (MLST), core genome MLST, and single-nucleotide polymorphism (SNP) analysis.

**Results:**

Three different MLST sequence types were identified and further subdivided into eight clusters. This approach conclusively confirmed officially reported primary and secondary outbreaks caused by the sale of infected animals. In individual cases, remarkable similarities were found between domestic and wild animal isolates, that is, differing by only 2–4 nucleotides. This similarity suggests brucellosis transmission events. Both wild boars and hares can be considered reservoirs of *Brucella* spp. infections, including brucellosis. In Northern Germany, persistent *B. suis* biovar 2 foci were detected, as well as transmission across Germany and potentially to other European countries. Notably, hare isolates varied significantly from the majority of German wild boar and domestic pig isolates.

**Discussion:**

As most brucellosis outbreaks occurred in outdoor holdings, reliable monitoring of these herds is recommended, although the exposure of these animals to external factors (e.g., vectors) poses a challenge. However, it is imperative in light of the increase in organic free-range and pasture farming, which promotes direct or indirect contact with wild animals.

## Introduction

Brucellosis, which is caused by various species of the genus *Brucella*, remains a major zoonosis worldwide, with over two million new human infections annually (1). *Brucella melitensis*, *Brucella abortus*, and *Brucella suis* are the most critical zoonotic *Brucella* species. The species exhibit different host preferences, with humans being accidental hosts who typically contract the infection through the consumption of contaminated food or direct contact with infected animals. Of the *B. suis* biovars, biovars 1 and 3 pose the highest risk to humans, while *B. suis* biovar 2 has a lower pathogenicity but can still cause infections in humans. Human infections with biovar 2 are rarely reported and mainly affect people with chronic diseases or patients undergoing therapies that weaken the immune system (2). Due to its zoonotic potential and its potential impact on public health and the economy, brucellosis is considered a One Health problem, particularly because of the difficult-to-control reservoirs in wildlife populations (3, 4).

The global prevalence of swine brucellosis is 2.1% (5). While *B. suis* biovar 1 and 3 occur in many regions of Asia, America, and Australia, there are only rare reports from European countries (6, 7). In contrast, *B. suis* biovar 2 is endemic and widespread in Europe (8–13). Here, the main reservoirs for *B. suis* biovar 2 are wild boar and hares (14, 15). The pathogen is often considered endemic in these animal species (16–18). The prevalence found in wild boar in Europe varies between regions and the period studied, reaching up to 55% (11–13, 19–21). However, some studies did not detect *Brucella*-specific antibodies in domestic pigs and wild boars, suggesting considerable differences in prevalence between regions (22, 23). Less information is available about *Brucella suis* biovar 2 in hares. Serological studies indicate that seropositivity rates in hares in Austria, Lombardy Region (Italy), and the Czech Republic were approximately 3.5, 0.2, and 1.6%, respectively (21, 24, 25). However, no positive hare was found in a study from Germany and the Czech Republic (26). It is worth noting that serological investigations may be biased by cross-reactions with antibodies other than those against *Brucella* sp., such as those against *Yersinia* sp. (27). Not all of the studies mentioned above checked for these cross-reactions. Thus, the actual prevalence in wildlife remains elusive.

Brucellosis was endemic in Germany until the 1980s (28) and the majority of human cases were caused by infection with *B. abortus*, the agent of bovine brucellosis. Most human brucellosis cases are now travel-associated, caused by *B. melitensis* (29). Germany achieved official brucellosis-free status for cattle through European Union Decision 1999/466/EC and for sheep and goats through Decision 93/52/EEC. This has been achieved through strict control and eradication programs and is maintained to this day through ongoing testing programs. The status is reassessed regularly by the Directorate General for Health and Food Safety (DG Sante) and European Food Safety Authority (EFSA), who receive information from EU Member States. However, pig herds are not subject to any general surveillance obligation regarding brucellosis. There are only testing regulations for insemination stations (30), as the venereal route is considered the main transmission route for porcine brucellosis. In contrast, cattle older than two years herds undergo serological testing every three years.

To date, the knowledge about porcine brucellosis at least in Germany is primarily based on serological investigations. In various studies, serological tests have been carried out on wild boar in different Federal States. In Mecklenburg-Western Pomerania, Bavaria, Lower Saxony, and Saxony, seroprevalence rates were found to be 22, 18, 35, and 21%, respectively (30–34). In Baden-Württemberg, however, only a maximum of 0.2% of samples were found to be positive (30, 35).

In addition to serological studies, pathological and bacteriological examinations of shot and fallen animals are carried out in suspected cases. In a survey conducted in Mecklenburg-Western Pomerania, which examined 888 wild boar samples, 14 testicular samples with typical pathological changes were identified. A total of 17 *Brucella* isolates were obtained from these samples, even from pathologically normal testicular tissues (36). Comparable results were obtained by examining rabbit carcasses (37, 38).

The majority of pigs produced in Germany are raised on intensive conventional farms. However, there are also free-range farms in climatically suitable regions that essentially practice organic farming, particularly in the north of the country.

In this study, we investigated *B. suis* isolates from 11 different German Federal States, obtained from both wild animals and domestic pigs. This study aimed to demonstrate the traceability of outbreaks on pig farms and to investigate whether a connection can be established between these outbreaks and wild animal reservoirs using a sequencing-based epidemiological approach. Further, this study gives the first comprehensive overview of the *B. suis* biovar 2 genotypes in Germany.

## Materials and methods

### Database search on brucellosis outbreaks

The German online database “Tierseuchen-Informationssystem” (TNS) was queried on 26 April 2024 for officially notified brucellosis outbreaks in Germany over the past 20 years.

### Isolate selection and cultivation

As part of its sovereign tasks, the National Reference Laboratory for *Brucella* sp. infections received suspected *Brucella* sp. isolates for analysis from the Federal State investigation offices. After routine diagnosis, the results were given to the responsible regional veterinary service, and the strains were stored long term in 80% glycerol in a strain collection. For the study, all available isolates were re-cultivated on nutrient agar (Merck KGaA, Darmstadt, Germany), supplemented with whole calf blood, at 37°C for 48 h.

### DNA isolation and identification by PCR

DNA was isolated using the High Pure PCR Template Preparation Kit (Roche Molecular Systems, Pleasanton, CA, United States). To confirm the identity as *Brucella* sp. and identify the species, Abortus, Melitensis, Ovis, Suis (AMOS) and Bruce-ladder polymerase chain reaction (PCR) were conducted as described before (39–41). Additionally, Suis-ladder PCR (42) was used for differentiation on the biovar level.

### Whole genome sequencing, quality control, and assembly

Sequencing libraries were prepared using the Nextera XT library preparation kit (Illumina Inc., San Diego, CA, United States). The libraries were sequenced on a MiSeq system in paired-end mode using v3 chemistry (Illumina Inc., San Diego, CA, United States) for 2 × 300 bp long reads. The quality of the raw sequencing reads was assessed by FASTQC v0.11.7^1^ and kraken2 v2.0.7_beta (43) for checking for contamination and confirming the species identity. Genomes were assembled in a *de novo* approach by Shovill v.1.0.4,^2^ which utilizes Spades as implemented in Shovill. The quality of the resulting assemblies was subsequently assessed by Quast v5.0.2 (44).

### *In silico* genotyping

To identify genotype clusters, Multilocus Sequence Typing (MLST) and core genome MLST (cgMLST) were conducted *in silico* based on the assemblies using mlst v2.19.0^3^ employing the public databases for molecular typing and microbial genome diversity (PubMLST) website (45) and Ridom SeqSphere+ v7.7 (46) applying the scheme by Abdel-Glil et al. (47), respectively. Following the cgMLST analysis, a minimum spanning tree was generated based on the allelic differences, as implemented in SeqSphere+ with pairwise ignoring missing values.

For a more detailed analysis, the clusters were individually subjected to core genome single-nucleotide polymorphism (cgSNP) analysis as an assembly-independent genotyping approach. In the SNP analysis, raw read data of *B. suis* deposited in the NCBI Sequence Read Archive (SRA) (accessed on 28 March 2023) were included (Supplementary Table 1). Sequences were chosen based on geographic origin and biovar., when available. The quality of this data was first controlled as described above.

SNPs were called by Snippy v.4.6.0^4^ in default mode with *B. suis* biovar 2 strain Thomsen/ATCC 23445 (GCF_000018905.1) as reference genome. The resulting core genome alignment was used as input for maximum likelihood analysis using RAxML v8.2.12 (48) with the GTR model (−m ASC_GTRCAT) without correction for rate heterogeneity (-V option) and ascertainment bias correction (--asc-corr= lewis), as recommended by the RAxML manual. The tree was visualized by Microreact (49). Further, SNP distance matrices were generated with snp-dists v0.7.0.^5^

## Results

### Database search

**Figure 1:**
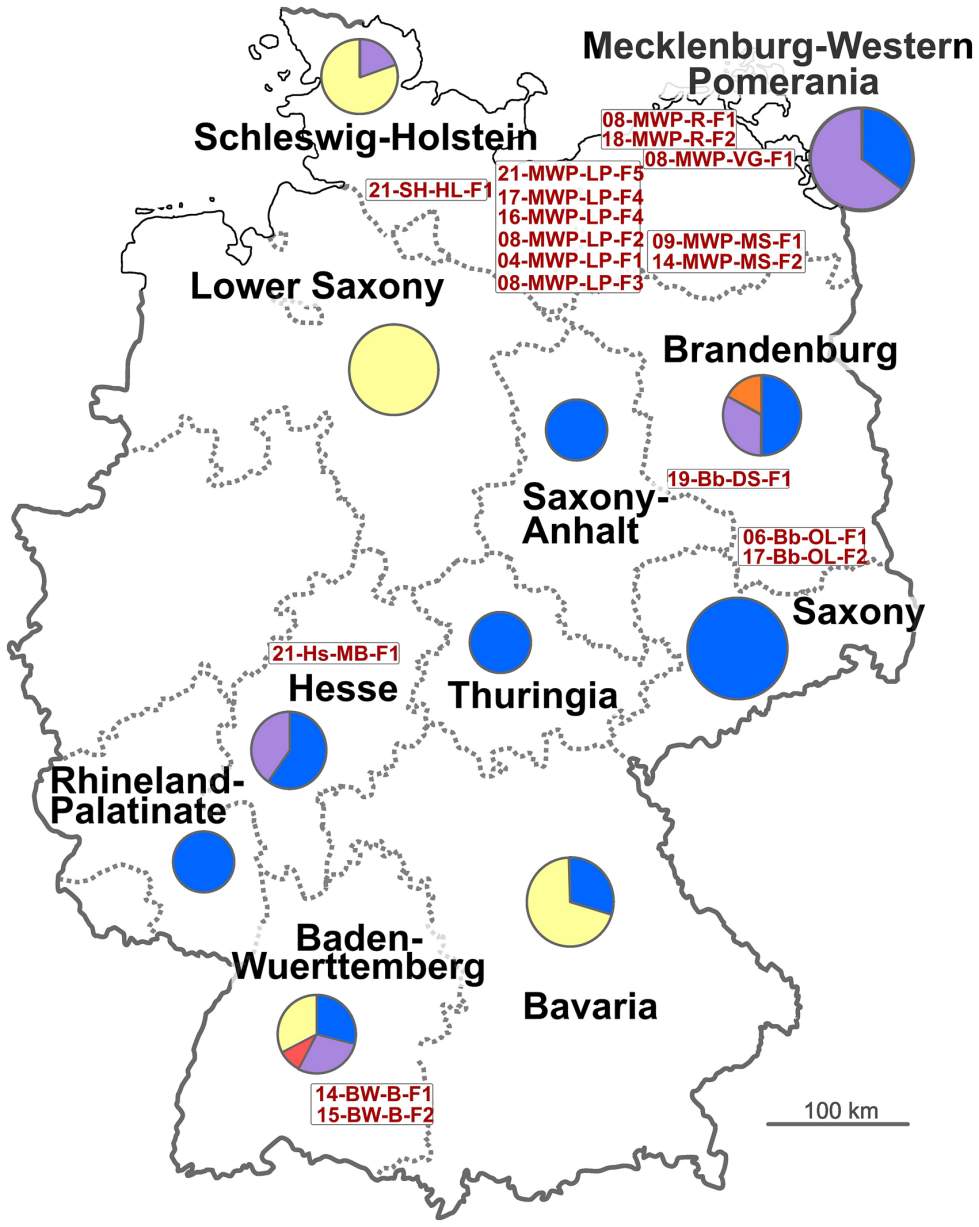
Map of Germany with relevant states. Circles represent numbers of isolates from the respective state, with colors indicating host (blue: *Sus scrofa*; violet: *Sus scrofa domesticus*; yellow: *Lepus europaeus*; orange: *Sus scrofa* mix; red: *Capreolus capreolus*). Written in red are the IDs of the notified brucellosis outbreaks (see Table 1).

Since 2003, 29 officially notified brucellosis outbreaks among domestic animals have been registered in the TierSeuchen-Nachrichtensystem (TSN). For most of them (*n* = 22), *B. suis* was identified as the causative agent. Furthermore, three outbreaks were reported as being caused by *B. melitensis*, *B. abortus*, and *B. ovis*, respectively. For four other outbreaks, the causative species was not determined. Of the 22 known *B. suis* outbreaks, four were exclusively confirmed by serological methods; therefore, they could not be considered in the current study. The majority of the remaining 18 brucellosis outbreaks (Table 1 and Figure 1) occurred in Mecklenburg-Western Pomerania (*n* = 11). For the majority of the outbreaks in other German Federal States, the source of infection was suspected to be the purchase of animals and contact with wild animals. In contrast, no source was identified for any of the incidents in Mecklenburg-Western Pomerania. There were two consecutive outbreaks in 2016 and 2017 on the same farm (LP-F4). All primary outbreaks, except 17-Bb-OL-F2, occurred on farms where pigs were kept outdoors (free-range farming).

**Table 1:** Officially notified brucellosis outbreaks in Germany between 2003 and 2023, from which isolates were obtained for analysis.

Year	State	District	Suspected infection source	Outbreak ID
2004	Mecklenburg-Western Pomerania^*^	Ludwigslust-Parchim	Unknown	04-MWP-LP-F1
2006	Brandenburg	Oberspreewald-Lausitz	Contact with wildlife	06-Bb-OL-F1
2008	Mecklenburg-Western Pomerania	Landkreis Rostock	Unknown	08-MWP-R-F1
2008	Mecklenburg-Western Pomerania	Ludwigslust-Parchim	Unknown	08-MWP-LP-F2
2008	Mecklenburg-Western Pomerania	Ludwigslust-Parchim	Unknown	08-MWP-LP-F3
2008	Mecklenburg-Western Pomerania	Vorpommern-Greifswald	Unknown	08-MWP-VG-F1
2009	Mecklenburg-Western Pomerania	Mecklenburgische Seenplatte	Unknown	09-MWP-MS-F1
2014	Mecklenburg-Western Pomerania	Mecklenburgische Seenplatte	Unknown	14-MWP-MS-F2
2015	Baden-Württemberg^**^	Biberach	Purchase of animals	15-BW-B-F1
2015	Baden-Württemberg	Biberach	Purchase of animals	15-BW-B-F2
2016	Mecklenburg-Western Pomerania	Ludwigslust-Parchim	Unknown	16-MWP-LP-F4
2017	Mecklenburg-Western Pomerania	Ludwigslust-Parchim	Unknown	17-MWP-LP-F4
2017	Brandenburg	Oberspreewald-Lausitz	Unknown	17-Bb-OL-F2
2018	Mecklenburg-Western Pomerania	Landkreis Rostock	Unknown	18-MWP-R-F2
2019	Brandenburg	Dahme-Spreewald	Purchase of animals	19-Bb-DS-F1
2021	Schleswig-Holstein^***^	Herzogtum Lauenburg	Unknown	21-SH-HL-F1
2021	Mecklenburg-Western Pomerania	Ludwigslust-Parchim	Unknown	21-MWP-LP-F5
2021	Hesse	Marburg-Biedenkopf	Purchase of animals	21-Hs-MB-F1

Outbreak identification (ID) is composed of year-state-district-farm. ^*^MWP, Mecklenburg-Western Pomerania; ^**^BW, Baden-Württemberg; ^***^SH, Schleswig-Holstein.

### Sample selection and identification by PCR

At least one isolate from each of the notified brucellosis outbreaks in Germany (Table 1) was obtained from the responsible Federal State investigation offices (Landesuntersuchungsamt) for molecular epidemiological investigation. A total of 106 German *B. suis* isolates were included in the analysis, that originated from 11 different states in Germany (Table 2, Figure 1, and Supplementary Table 2) over a 19-year period (2004–2023). The German isolates had been recovered from four different animal hosts, primarily from wild boar (*Sus scrofa*), but also from hare (*Lepus europaeus*), domestic pigs (*Sus scrofa domesticus*), a wild boar mix (a crossbreed of pig and wild boar), and from deer (*Capreolus capreolus*). Samples from domestic pigs were exclusively obtained from outdoor farms, mainly in Mecklenburg-Western Pomerania in the North-East of Germany, with one exception (17-Bb-OL-F2). Particularly, *B. suis* isolates from wild boars were retrieved from most of the studied states. In contrast, from Lower Saxony, only isolates from the hares were available.

**Table 2:** Number of German *Brucella suis* biovar 2 isolates included in the analysis, according to state of origin and host.

State	Host					Sum
	Wild boar	Domestic pig	Wild boar mix	Hare	Deer	
Baden-Württemberg	2	2		2	1	7
Bavaria	3			7		10
Brandenburg	3	2	1			6
Hesse	3	2				5
Lower Saxony				13		13
Mecklenburg-Western Pomerania	10	17				27
Rhineland-Palatinate	2					2
Saxony	27					27
Saxony-Anhalt	2					2
Schleswig-Holstein		1		4		5
Thuringia	2					2
**Sum**	**54**	**24**	**1**	**26**	**1**	**106**

PCR analysis confirmed the identity of the isolates as *B. suis.* All investigated strains were further identified as *B. suis* biovar 2.

### Assembly-based genotyping

*De novo* assembly of the sequenced strains yielded genomes composed of 25–43 contigs with a GC content of 57.18–57.24% and sizes ranging between 3,232,502 and 3,324,607 bp with a mean N50 value of 193 kb. The mean read coverage was 169, ranging between 67 and 570. These values accounted for sufficient sequencing depth and quality. Thus, all isolates could be included in the downstream analysis. Based on the assemblies, the isolates were initially classified *in silico* by classical MLST-9 analysis. Three different sequence types (ST) were identified. Most isolates (*n* = 67) belonged to ST16. Further, ST15 (*n* = 33) and ST104 (*n* = 8) were found. While ST16 and ST104 were primarily found in wild boars and domestic pigs, ST15 was dominated by isolates from hares (79%) in our dataset.

**Figure 2:**
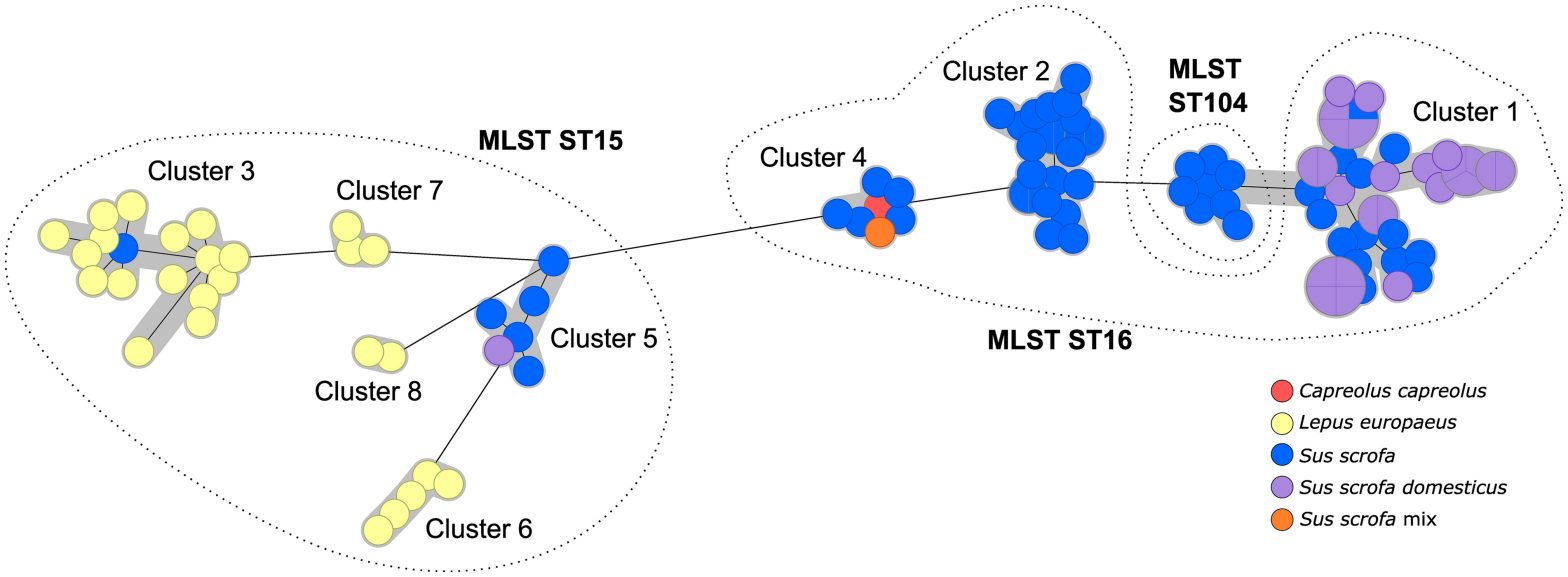
Minimum spanning tree based on cgMLST allelic distances of 106 German *Brucella suis* biovar 2 isolates. Clusters are indicated by gray shading. Leaf colors give the host from which the isolate originated. Dashed lines show affiliation of cgMLST clusters to the corresponding MLST sequence type (in bold).

These sequence types could be further subdivided by cgMLST to generate clusters that can then be investigated in detail by SNP typing. In all isolates, 95.9–100% of the target loci were identified (“good targets”), with a mean value of 99.7% good targets. A cut-off of 100 alleles was chosen for cluster definition to optimize the number of strains within one cluster, facilitating follow-up investigations. Thus, eight different cgMLST clusters were identified (Figure 2), which divided ST15 into five and ST16 into three clusters. The isolates of ST104 were part of the cgMLST Cluster 1. Notably, the cgMLST analysis confirmed the tendency found in MLST analysis, as these clusters comprised almost exclusively isolates from one host type: either hares or wild boars/domestic pigs. A distinct connection to the geographic origin of the isolates, on the other hand, was not observed. The clusters were separated by at least 102 alleles.

### SNP analysis of cgMLST clusters

To investigate the clusters defined by cgMLST in more depth, each cluster was subjected to cgSNP typing separately. In this way, minute differences between isolates could be resolved to investigate epidemiological links. The results for each cluster are given separately. Only the clusters that belonged to MLST ST15 were analyzed together, for conciseness.

### SNP typing of cgMLST Cluster 1

**Figure 3:**
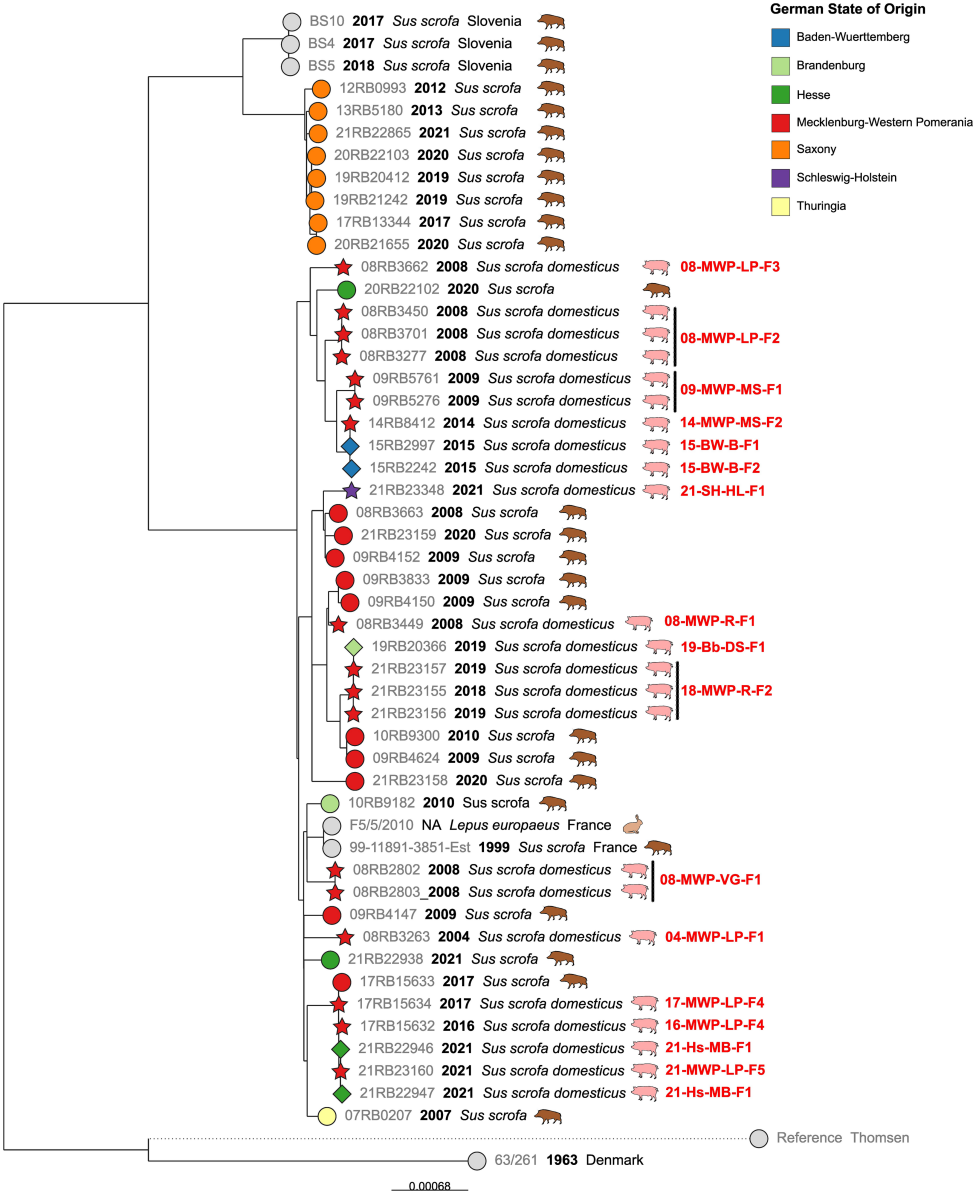
Maximum likelihood tree based on cgSNP alignments of German *Brucella suis* biovar 2 isolates of cgMLST Cluster 1. Leaf colors indicate the German state of origin. Foreign strains are colored gray. Leaf shapes indicate the type of isolate (circle: wildlife or unknown; pentagram: notified outbreak without known source; diamond: notified secondary outbreak). Leaf labels give strain name, year of isolation, and host. Outbreak identifications (IDs) are shown in red after corresponding isolates.

By far, the largest cluster was the cgMLST Cluster 1, comprising 45 German isolates. In detail, this cluster comprised isolates from most of the notified brucellosis outbreaks, particularly from Mecklenburg-Western Pomerania (Figure 3).

From four brucellosis outbreaks among domestic pigs (08-MWP-VG-F1, 08-MWP-LP-F2, 09-MWP-MS-F1, and 18-MWP-R-F2), two to three isolates from different animals per outbreak were available. The intra outbreak cgSNP difference was at a maximum of two SNPs. However, for three of these outbreaks, all isolates were identical in terms of cgSNPs (0 SNP difference).

The earliest outbreak, represented by a single isolate, was reported in 2004 in the district of Ludwigslust-Parchim, Mecklenburg-Western Pomerania (04-MWP-LP-F1). This isolate differed in at least 41 SNPs from others. Four years later, in 2008, four brucellosis outbreaks were officially reported in three provinces of Mecklenburg-Western Pomerania (Table 1), and at least one isolate of each was included in the present analysis, all of which were contained in Cluster 1. Isolates from these outbreaks did not form a single cluster. Although 08-MWP-LP-F2 and 08-MWP-LP-F3 occurred in the same district in 2008, the isolates differed in 39 to 40 SNPs. Furthermore, the difference in the other two outbreaks (08-MWP-VG-F1 and 08-MWP-R-F1), which differed in 46 SNPs, was on average 51 SNPs.

In the following years, more outbreaks were reported in the same state, whose corresponding isolates belonged to the same cgMLST cluster. In 2009, Mecklenburgische Seenplatte district, an outbreak (09-MWP-MS-F1) occurred, whose isolates belonged to the same monophyletic group as those of outbreak 08-MWP-LP-F2, but still differed in 28 to 29 SNPs from the latter. Five years later, an outbreak occurred among outdoor farm pigs in the same district (14-MWP-MS-F2), with an isolate that differed in 19 SNPs from those of 2009. Additionally, in early 2015, two outbreaks were reported on two farms in a different state, Baden-Württemberg (15-BW-B-F1, 15-BW-B-F2), which could both be traced back to the 2014 outbreak in Mecklenburg-Western Pomerania by the authorities. The 2015 isolates were identical or differed in one SNP from the 2014 isolate, respectively.

A similar incidence of transmission of an outbreak strain from Mecklenburg-Western Pomerania to a different Federal State was observed in 2018/2019. Isolates from an outbreak in the district of Rostock from late 2018 to early 2019 (18-MWP-R-F2) were identical in cgSNPs to an isolate that originated from a known secondary outbreak on a farm in Brandenburg (19-Bb-DS-F1), a state bordering Mecklenburg-Western Pomerania to the south. These outbreak isolates exhibited 17 SNP differences to two wild boar isolates from Mecklenburg-Western Pomerania in 2009 and 2010.

Notably, one branch comprised isolates from four different outbreaks within a 5-year period, whose genomes were almost identical (0–4 SNPs difference). The first of these outbreaks, 16-MWP-LP-F4, was reported in June 2016, followed by a second outbreak on the same farm one year later (17-MWP-LP-F4). Furthermore, four years later, in the same district but at a different farm, an isolate with the same cgSNP genotype was isolated during outbreak investigations (21-MWP-LP-F2). Further, isolates from an outbreak in Hesse, notified one month later (21-Hs-MB-F1), also exhibited the same genotype. For the later outbreak, it had been noted that the purchase of animals was a contributing factor. Remarkably, this cluster of outbreak isolates also included one wild boar isolate from Mecklenburg-Western Pomerania in 2017, which differed in 2–4 SNPs from the farm animals.

Besides the outbreak strains, cgMLST Cluster 1 also included isolates from wild boar. Notably, the wild boar isolates from Saxony, which were the only isolates with ST104, formed a single branch, separate from the other isolates in this cluster, displaying a maximum of 18 SNPs difference from each other. A neighboring branch comprised strains from Slovenia, also from wild boar, which differed in 71–77 SNPs from the Saxon isolates. No Saxon isolates were present in the larger branch of the polytomy, although it included wild boar isolates from neighboring as well as more distant German states. Wild boar isolates from other states exhibited predominantly larger differences. Two isolates from Hesse differed in 48 SNPs. The sole Thuringian isolate in this cluster displayed at least 30 SNPs’ difference from other isolates, and the Mecklenburg-Western Pomeranian isolates differed in 11–60 SNPs.

### SNP typing of cgMLST Cluster 4

**Figure 4:**
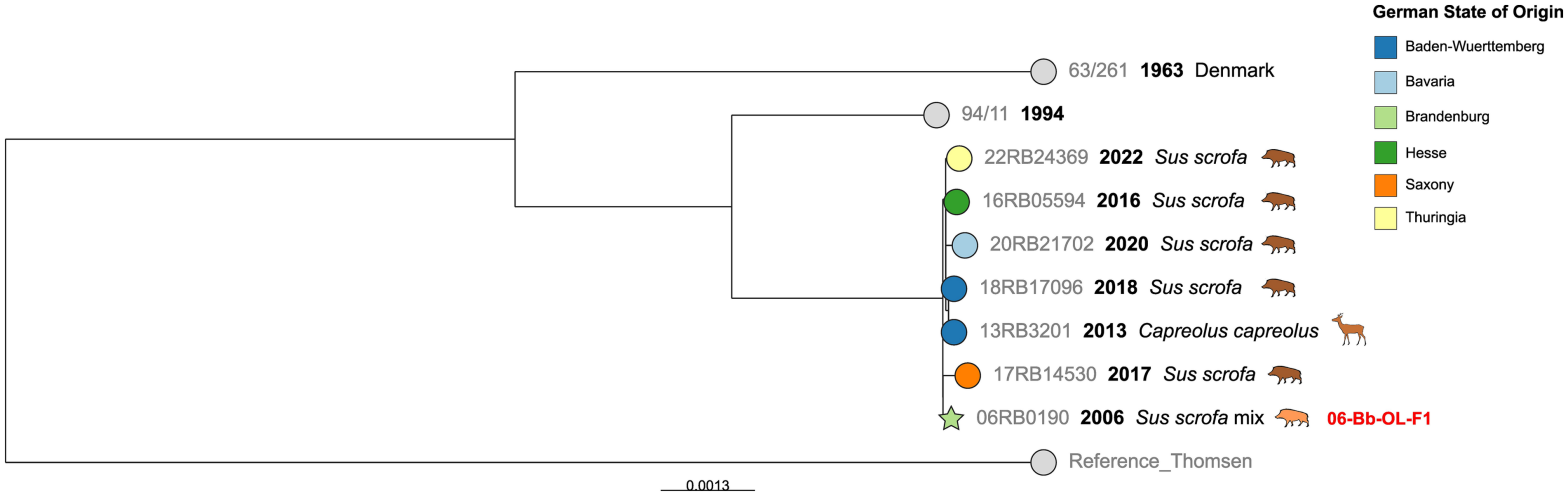
Maximum likelihood tree based on cgSNP alignments of German *Brucella suis* biovar 2 isolates of cgMLST Cluster 4. Leaf colors indicate the German state of origin. Foreign strains are colored gray. Leaf shapes indicate the type of isolate (circle: wildlife or unknown; pentagram: notified outbreak without known source). Leaf labels give strain name, year of isolation, and host. Outbreak IDs are displayed in red next to the corresponding isolates.

Cluster 4 was composed of seven German isolates from six different Federal States (Figure 4). Despite its geographic heterogeneity, the maximum number of other SNPs within this cluster was 17, although the isolates were obtained up to 16 years apart. Furthermore, this cluster contained the only isolate from deer (*Capreolus capreolus*) in the investigated dataset, isolated in Baden-Württemberg in 2013, which was almost identical to an isolate from wild boar from the same region from 2018 (four SNPs difference). An isolate from the Brandenburg outbreak 06-Bb-OL-F1 also fell within this cluster, displaying 7 to 12 SNPs difference from the wild *Sus scrofa* isolates. Notably, contact with wildlife had been suspected as the source of infection for this outbreak by the authorities.

### SNP typing of cgMLST Cluster 2

**Figure 5:**
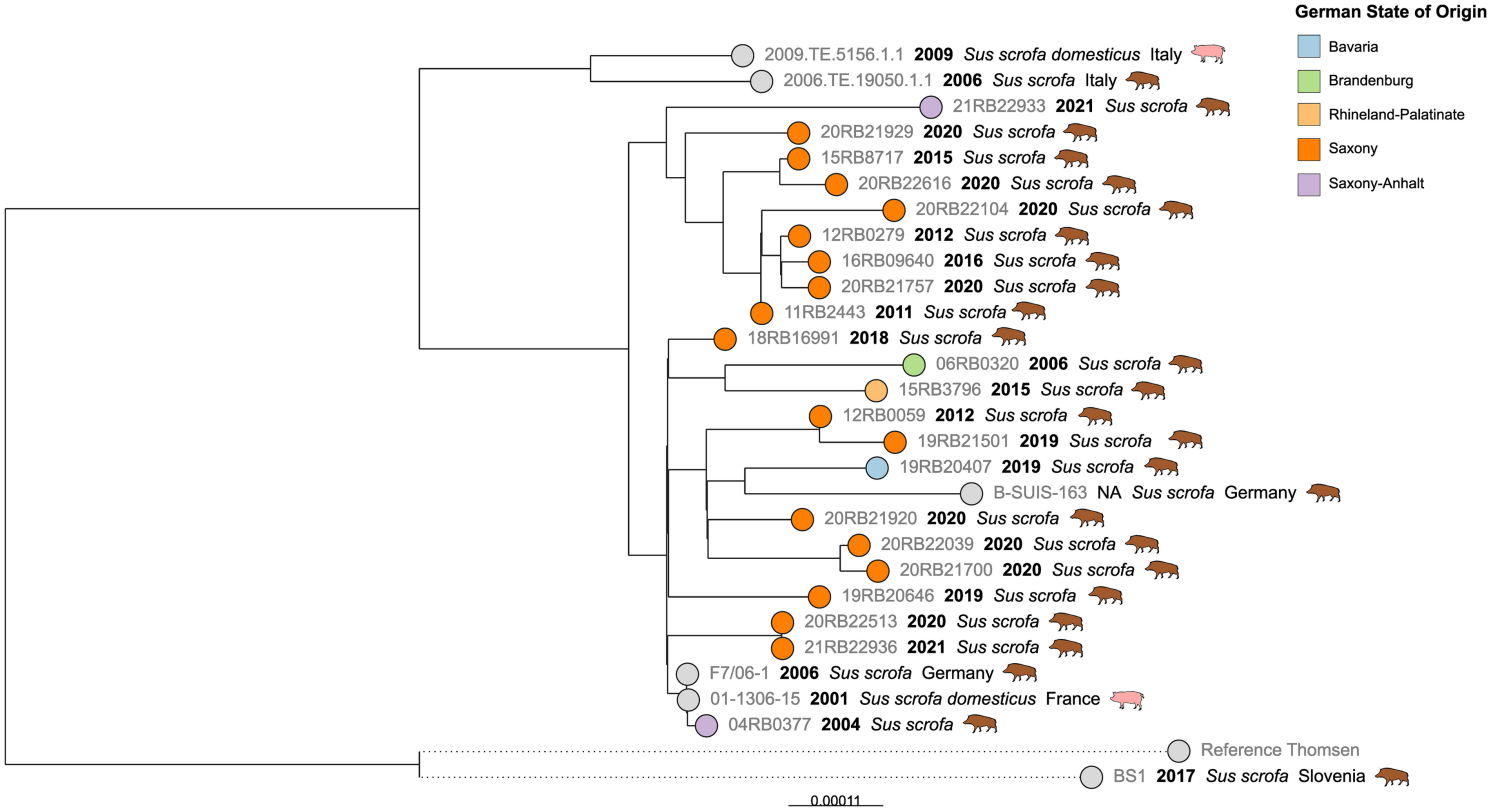
Maximum likelihood tree based on cgSNP alignments of German *Brucella suis* biovar 2 isolates of cgMLST Cluster 2. Leaf colors indicate the German state of origin. Foreign strains are colored gray. Leaf labels display the strain name, year of isolation, and host.

The German isolates in Cluster 2 were exclusively isolated from wild boar, predominantly from Saxony, over a 17-year period (2004–2021) (Figure 5). SNP variations between the isolates were low, for example, ranging from 2–28 SNP differences within the Saxon population. The only two wild boar isolates from Saxony-Anhalt, isolated in 2004 and 2021, differed in 20 SNPs. Notably, a strain from France, isolated from *Sus scrofa domesticus* in 2001, differed in a single SNP from a German wild boar isolate from Saxony-Anhalt found three years later. The difference to the Saxon isolates was 4–13 SNPs.

### SNP typing of MLST ST15 clusters

**Figure 6:**
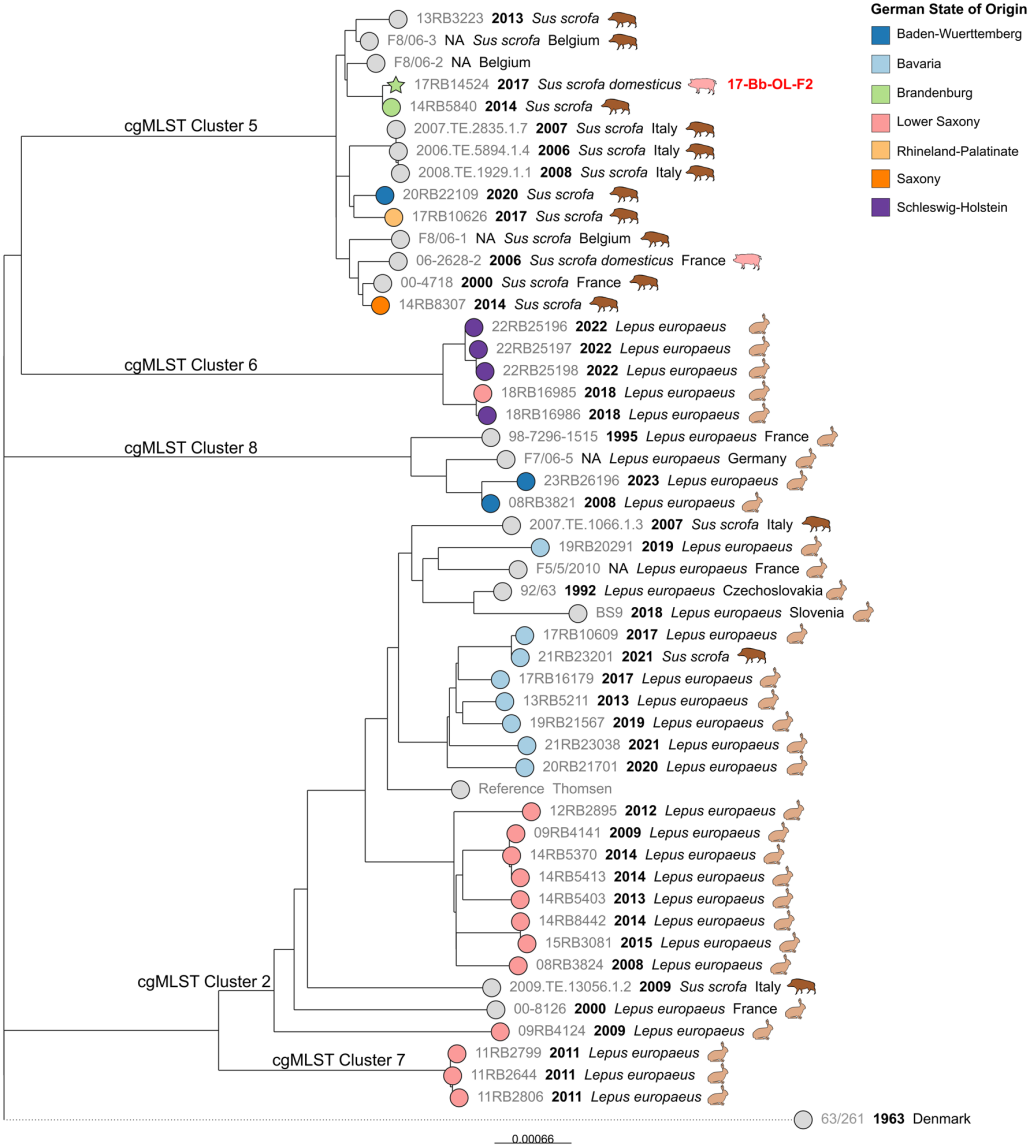
Maximum likelihood tree based on cgSNP alignments of German *Brucella suis* biovar 2 isolates of cgMLST Clusters 3 and 5–8. Leaf colors indicate the German state of origin. Foreign strains are colored gray. Leaf shapes indicate the type of isolate (circle: wildlife or unknown; pentagram: notified outbreak without known source). Leaf labels give strain name, year of isolation, and host. Outbreak identification (ID) is shown in red after the corresponding isolate.

The cgMLST Clusters 3, 5, 6, 7, and 8, which all belonged to MLST ST15, were analyzed together (Figure 6). The cgSNP analysis confirmed that the hare isolates formed clusters, separate from other wildlife isolates, with one exception: one isolate obtained from a wild boar in 2021 in Bavaria exhibited 10 SNPs difference from a hare isolate from the same state that was isolated four years before. Interestingly, the majority of foreign strains included in the analysis were also isolated from hares in various European countries.

In contrast to the Bavarian hare isolates, which formed a separate branch within cgMLST Cluster 3, isolates from Lower Saxony were found on different branches. However, the isolates from both states originated from a comparable time frame spanning eight and ten years, respectively. Likewise, four isolates from Schleswig–Holstein formed a separate branch, which also comprised one hare isolate from Lower Saxony, that displayed eight SNP differences to an isolate from the same year. Two hare isolates from Baden-Württemberg formed a distinct cluster with another German isolate and a French isolate from 1995, both of which originated from hares.

Separated from the hare isolates by more than 358 SNPs, a branch was formed by *Sus scrofa* isolates from Belgium, Italy, and France, as well as wild boar isolates from four different districts of Germany. Notably, an outbreak isolate from a domestic pig in Brandenburg in 2017 (17-Bb-OL-F2) was also included in this cluster and exhibited 10 SNP differences to a wild boar isolate from the same state, isolated three years prior. Both isolates differed from an isolate from Belgium in 33 and 38 SNPs, respectively. The difference between a French isolate from 2014 and a wild boar isolate from Saxony, a Federal State located in eastern Germany, was merely 17 SNPs.

## Discussion

The German *Brucella* isolates analyzed in this study originated from samples routinely submitted to the NRL for brucellosis in cattle, pigs, sheep, and goats. The detection of brucellosis caused by *B. melitensis*, *B. abortus*, and *B. suis* is notifiable in Germany. The National Animal Disease Notification System (TSN) is used to record reported outbreaks. Of the 29 brucellosis outbreaks registered in the TSN between 2003 and 2023, 18 outbreaks could be traced back to infections with *B. suis* biovar 2. To facilitate comparison with wild animal isolates in the present study, isolates identified as *B. suis* biovar 2 from wild boar, hares, and a deer from the same period were included. Genomes of all isolates were sequenced and bioinformatically analyzed.

All 106 isolates were initially classified using *in silico* MLST-9. Three sequence types were identified. Most of the wild boar isolates and all but one of the outbreak isolates belonged to the ST16, which has previously been described in isolates from France and Croatia (50). Isolates of this sequence type were also found in Slovenian and Italian wild boar (51, 52). It is a single-locus variant of ST15, which is the sequence type of the reference strain Thomsen (50). All of the investigated hare isolates, a few wild boar isolates, and only one outbreak isolate belonged to ST15. By this result, a study from Bavaria also found only sequence types 15 and 16 in a total of five *B. suis* isolates from wild boar isolated between 2019 and 2021 (32). The majority of isolates of ST15 described to date originated from wild boar, but there are also a few from hares (45). Furthermore, in our study, ST104 was only found in wild boar. To our knowledge, this sequence type has so far only been identified in wild boar in Slovenia (51).

The resolution of the MLST-9 and the MLST-21, scheme is known to be insufficient for carrying out precise analyses of the genotypes of individual isolates (53). Therefore, we performed cgMLST analysis to further subdivide the isolates and identified eight clusters, which were then analyzed in detail.

CgMLST and cgSNP typing are commonly applied to investigate outbreaks and epidemiological links. In cgMLST, allelic profiles of genes are generated, whereby multiple nucleotide changes in a locus are reduced to a single allelic number shift, thereby simplifying the complexity. SNP typing, on the other hand, provides a more detailed insight into base substitutions, as it includes intergeneric regions and considers all changes. Nevertheless, many laboratories use cgMLST for outbreak investigations (54).

Interestingly, the result of the MLST analysis was confirmed by the clustering of the strains in cgMLST analysis, insofar as the hare and pig isolates were essentially placed in different clusters. Additionally, the hare isolates clustered more closely according to their geographic origin. Such observations have already been made based on multilocus variable-number tandem repeat analysis (MLVA) and SNP analyses (16): the authors identified different clades which were in accordance with their geographic origin, with hares belonging to two clusters of the Central European clade. Hare isolates also differed from wild boar isolates in MLVA investigations in Hungary (18). In another study, *Brucella* isolates with similar MLVA profiles were obtained from both animal species (55). It can be assumed that although hares share the same habitat with other wildlife, they do not have particularly close contact with wild boars. Thus, pathogen transmission could be impeded. The observed examples of high genetic similarity of *Brucella* isolates from both animal species are probably rare occurrences of possible direct pathogen transmission. These results are consistent with published data on the relatively limited habitats of hares compared to wild boar (56, 57). Furthermore, the different sequence types in isolates from wild boar and hares might also represent a host specificity of *B. suis* biovar 2 MLST ST15. However, more data are required to substantiate this hypothesis and assess the role of hares in the transmission cycle of porcine brucellosis.

The cgMLST clusters were further analyzed in terms of their cgSNP differences. In Cluster 1, the largest of the observed cgMLST clusters, a total of 45 German isolates comprising 22 wild boar isolates and 23 outbreak isolates, were identified from 17 of the 19 outbreaks that occurred during the investigated period. Several isolates were available from each of the four outbreaks, allowing for the investigation of intraoutbreak SNP differences. The sequences of isolates from the same outbreak were either identical or differed by a maximum of two SNPs in the cgSNP typing. This demonstrates the high reliability of sequencing results in relation to possible sequencing errors. It can also be assumed that each outbreak was caused by a single *B. suis* biovar 2 genotype, that is, from a single source of infection.

From the German Animal Disease Notification System (TSN), information on the nature of the outbreak, i.e. whether it was a primary or secondary outbreak, was obtained. Further, the identified reasons for secondary outbreaks were stated in this database. Secondary outbreaks were caused by the purchase of animals from other farms, which raises the question of why infected animals could be sold. In one case, this was a shortcoming of the state veterinarian, who misinterpreted the applicable legal provisions and moved animals under four months to other herds (personal communication). Under German law, these animals are not included in outbreak investigations because they are not yet considered fully immunocompetent. However, they cannot be regarded as free of *Brucella* infection. This misjudgment underscores the importance of raising awareness about the epidemiology of brucellosis among veterinarians, for example, through regular training on surveillance practices. In the second primary outbreak case, animals were sold a few days before the *Brucella* infection was detected in the original herd. As part of traceability investigations, these animals were later diagnosed as infected and reported as secondary outbreaks. The sequences of the isolates from corresponding primary and secondary outbreaks were identical or differed only in a maximum of one SNP. With these results, epidemiological links between outbreaks can be proven beyond doubt.

The isolates in cgMLST Cluster 1 primarily originated from Mecklenburg-Western Pomerania, suggesting that this genotype is a persistent, dominant lineage in Northeast Germany, as it was found repeatedly between 2004 and 2024. Outbreaks 09-MWP-MS-F1 and 14-MWP-MS-F2, for example, which occurred five years apart but in the same district, were caused by the same *B. suis* biovar 2 lineage, that is, isolates differed in 19 cgSNPs, indicating genetically stable brucellosis foci. Since the overall differences between the wild and domestic pig isolates, as well as between the outbreaks themselves, were not high, particularly considering the period, it can be assumed that there were frequent transmissions between wild and domestic animals. Accordingly, Keuling, et. al. (58) found that in Mecklenburg-Western Pomerania, in the same region from which most isolates of cgMLST Cluster 1 originated, wild boars predominantly did not leave their home range (natal area). This could allow for the stabilization of brucellosis foci. A transmission between wild animals and pigs can be assumed for outbreak 17-Bb-OL-F2 from the cgMLST Cluster 5, as its isolate was highly similar in cgSNP to a wild boar isolate from the same federal state. Other studies have shown that contact between domestic pigs and wild boars is challenging to prevent, particularly when the animals are reared outdoors (10, 59). In Saxony, a study reported brucellosis cases on a pig farm in 1994 following the birth of pig-boar hybrids three months prior, from which also *B. suis* biovar 2 was isolated (34).

The high similarity between isolates from different farms (21-MWP-LP-F2 and 16-MWP-LP-F4) even indicated a transmission between two domestic herds via an unknown vector.

The fact that the isolates from outbreaks 17-MWP-LP-F4 and 16-MWP-LP-F4 on the same farm differed merely in individual SNPs suggested that the pathogen persisted in the herd, despite the brucellosis control measures taken. In the event of a brucellosis outbreak in Germany, the requirements of the Brucellosis Ordinance must be followed. This requires all animals in the outbreak herd to be serologically tested, except for those younger than four months. Positive animals must be removed from the herd. Apparently, in the case of outbreak 16-MWP-LP-F4, not all infected animals have been removed; i.e. there were asymptomatic carriers, or an additional *Brucella* reservoir may have existed. It could be assumed that individual animals may show false negative results or that young pigs are already infected and therefore remain in the herd. These may pass on the infection. Some authors assume that, depending on the husbandry conditions, transmission from wild animals to domestic pigs, which are kept outdoors, can occur. If pigs are kept under high safety standards, such as double fencing, this transmission route is rather unlikely. Other animate and inanimate vectors come into question here (60, 61), as *B. suis* can also infect different hosts, for example, dogs and rats (62–65), which could pass the infection on. However, *B. suis* can also persist in the environment, for example, on concrete, particularly at low temperatures (66).

In cgMLST Cluster 4 was a previously described *B. suis* biovar 2 isolate from a deer (67). This showed only four cgSNPs difference to an isolate from a wild boar from the same Federal State. Other very similar genotypes from different years and neighboring Federal States can also be found in this cluster, suggesting that this genotype persists in southern and central Germany, as seen by other studies (32).

Looking at the *B. suis* biovar 2 diversity in the different Federal States, it was noticeable that there was at least one wild boar isolate from Saxony in each cgMLST cluster, except for the hare isolate-dominated clusters. The majority of Saxon wild boar isolates were found in the cgMLST Cluster 2, suggesting a dominant genetic lineage. Since the allelic differences between isolates were high (>100), this diversity in Saxony can be attributed to animal movement, probably also across the borders to Poland and the Czech Republic. A comparison of *B. suis* biovar 2 genotypes in neighboring regions would help elucidate the dynamics of regional porcine brucellosis transmission. Interestingly, the wild boar isolates from Saxony in the cgMLST Cluster 1 clustered with isolates from the Slovenian wild boar rather than German isolates. Further, isolates from other European countries were also found in all clusters, indicating a mixing of wild boar populations in relatively large geographical areas. It is known that wild boars can migrate long distances during their lifetime (68–70). Additionally, resettlements of wild boar could lead to the establishment of their pathogen’s genotypes in distant regions (37, 71, 72).

In Germany, almost exclusively free-range holdings are affected by *B. suis* biovar 2. Wild boar, hares, and, to a lesser extent, other wild animals serve as reservoirs for the pathogen. However, direct contact between these animals and domestic pigs is not the only route of infection. Transmission between farms can occur through the trade of animals from already infected herds. Therefore, indoor holdings can also be affected. The existing tendency to prefer organic animal husbandry, including outdoor or pasture-based husbandry, can increase the risk of infection not only with *Brucella* spp. from wild animals, but also with other pathogens. Additionally, studies predict that climate change could lead to an increase in the wild boar population in central Europe (73). Therefore, appropriate monitoring measures must be implemented to address this situation, as no systematic surveillance of brucellosis in wildlife is currently carried out in Europe. This monitoring should take into account that symptom-free animals might act as carriers for brucellosis, and brucellae can even be isolated from apparently healthy animals (74). Therefore, molecular diagnostics, including WGS, must be an integral component of the surveillance system. Concerted, Europe-wide monitoring of the wildlife population is desirable, not only for monitoring the brucellosis situation, but also to enable a prompt response to developments. Here, also rarely investigated reservoirs, like deer, should be included. Such a cross-border One Health initiative can help elucidate the role of reservoirs and the dynamics of the *B. suis* population.

## Data Availability

The raw read data of the presented dataset were uploaded to the European Nucleotide Archive (ENA) and can be accessed under BioProject PRJEB61768.

## Footnotes

^1^ https://www.bioinformatics.babraham.ac.uk/projects/fastqc/

^2^ https://github.com/tseemann/shovill

^3^ https://github.com/tseemann/mlst

^4^ https://github.com/tseemann/snippy

^5^ https://github.com/tseemann/snp-dists
